# A Mixed Methods Approach to Understanding Mental Health Literacy Among University Health Students

**DOI:** 10.3390/healthcare13070724

**Published:** 2025-03-25

**Authors:** Ana Isabel Teixeira, Sónia Martins, Sara Lima, Francisca Pinto, Tânia Morgado, Olga Valentim

**Affiliations:** 1 Nursing School of Tâmega e Sousa, IPSN-CESPU, 4560-485 Penafiel, Portugal; sara.lima@ipsn.cespu.pt (S.L.); francisca.pinto@ipsn.cespu.pt (F.P.); 2 RISE-Health, Nursing School of Porto (ESEP), 4200-072 Porto, Portugal; tmorgado@gmail.com; 3 iHealth4Well-Being—Innovation in Health and Well-Being—Research Unit, IPSN-CESPU, 4560-485 Penafiel, Portugal; sonia.vmartins@cespu.pt; 4 UCIBIO—Applied Molecular Biosciences Unit, Translational Toxicology Research Laboratory, University Institute of Health Sciences (1H-TOXRUN, IUCS-CESPU), 4585-116 Gandra, Portugal; 5 RISE—Health, Faculty of Medicine, University of Porto, 4200-319 Porto, Portugal; 6 ISSSP—Higher Institute of Social Services of Porto, 4460-362 Porto, Portugal; 7 Pediatric Hospital of Health Local Unit of Coimbra, 3004-561 Coimbra, Portugal; 8 Health Sciences Research Unit: Nursing (UICISA: E), Nursing School of Coimbra (ESEnfC), 3045-043 Coimbra, Portugal; 9 Nursing School of Coimbra, 3004-011 Coimbra, Portugal; 10 Nursing School of Lisbon (ESEL), 1600-096 Lisboa, Portugal; 11 Nursing Research, Innovation and Development Centre of Lisbon (CIDNUR), Nursing School of Lisbon (ESEL), 1600-096 Lisboa, Portugal

**Keywords:** university students, mental health, mental health literacy, health promotion, mixed methods

## Abstract

Mental health literacy (MHL) is defined as the ability to obtain, understand, and use information to identify, manage, and prevent mental health problems and helps university health students to manage mental health challenges by reducing stigma, fostering resilience, and promoting coping strategies. **Objectives**: To assess MHL levels in Portuguese university health students and explore its relationship with academic life and psychosocial domains; Identify knowledge gaps and educational needs regarding a targeted MHL intervention. **Methods**: A convergent parallel mixed-methods study, involving collecting quantitative (online cross-sectional survey) and qualitative (focus group) data simultaneously, followed by a separate and integrative analysis. **Results**: Twenty-four students (75% female, mean age = 25.5) were included. Overall, differences were found in the MHL domains in terms of sex (*p* = 0.025), mental health history variables (*p* = 0.020; *p* = 0.044; *p* = 0.007), and a negative relation with academic environment satisfaction (r_s_ = −0.571). Focus groups revealed the needs, gaps, and main characteristics for a targeted intervention. Integrative analysis used for data triangulation was possible and helped to converge and reinforce some of the data findings. **Conclusions**: This study highlights the importance of integrated methodological approaches to explore MHL among undergraduate health students. Also, it highlights the importance of promoting MHL through targeted interventions to enhance well-being and reduce distress in academic contexts.

## 1. Introduction

Mental health literacy (MHL) is widely recognized as essential for promoting psychological well-being, especially in higher education settings. MHL is defined by Jorm et al. as the ability to obtain, understand, and use information to identify, manage, and prevent mental health (MH) problems [1]. Included in this definition is the recognition of the symptoms of psychological disorders, knowledge of their main causes and risk factors, attitudes regarding MH, and the ability to access both mental information and professional services [2]. This concept has been highlighted as crucial for improving emotional well-being, reducing stigma, and fostering help-seeking behaviors. MHL plays an important role in the recognition of MH problems and having the ability and knowledge to seek help for them [2,3,4], and a low MHL level has been associated with compromised MH outcomes, particularly higher levels of depression and anxiety [2,5,6].

Some authors have identified contributors to MHL in university students, such as: (1) coursework: students from health-related fields having improved knowledge regarding their curriculum; (2) experience: those who have MH issues and have been diagnosed and/or treated or have a family member with a MH disorder and also have a different MHL level. Demographic variables, including gender, age, and ethnicity, significantly contributed to differences in MHL scores [2].

In recent years, the concept of positive MHL (PMeHL) has gained relevance, broadening the traditional perspective to include skills such as resilience, self-efficacy, and constructive problem-solving. PMeHL goes beyond understanding MH problems, encompassing skills that promote well-being, personal fulfillment, and the ability to form social support networks [7]. This model is particularly pertinent in higher education, where promoting positive mental health acts as a protective factor against stress and academic pressures, and students with higher scores of positive mental health have higher levels of MHL [8,9].

Its relevance is particularly notable among students in the health field, who face rigorous academic demands and have the future responsibility of promoting MH in the communities where they work [7,10]. In higher education, a phase characterized by intense personal and academic transitions, MHL plays a central role, enabling students to manage academic pressures and social isolation and adapt to new routines [8,11].

Recent studies have demonstrated that high levels of MHL are associated with better emotional, academic, and social outcomes. Students with higher MHL have a greater ability to identify signs of psychological distress and implement effective coping strategies [12].

Structured programs [12,13,14] show effectiveness in promoting MHL and reducing stigma, especially in students in the health field.

Furthermore, some authors have emphasized the need to integrate digital technologies, such as mobile applications, to improve the accessibility and effectiveness of MHL programs in young populations. Digital MHL interventions are as effective as face-to-face in improving MHL and enhancing MH functioning [15]. However, privacy concerns regarding one’s information being accessible to third parties remain among higher education students [16].

Despite advances, gaps persist in understanding how students perceive and value MHL. Despite the range of mental health services available on campus, uptake of MH services among students is still poor. Recent studies have reported that the reason for the lack of help-seeking included MH stigma attitudes or a perceived lack of need. Students tend to seek help from informal sources, such as friends and family [17]. On the other hand, the authors reinforced the importance of integrating dimensions, such as happiness and personal fulfillment, in the development of MHL programs, highlighting their relationship with psychological well-being and academic satisfaction [18]. Other studies have reinforced the importance of understanding the demographic and educational factors that shape university students’ MHL and their attitude towards seeking psychological help, where targeted interventions addressing these factors can play a critical role in fostering positive MH behaviors [19]. Therefore, investment in MH education for university students is crucial [17].

Psychological well-being, based on the notion of eudaimonia proposed by Carol Ryff [20,21], conceptualizes psychological well-being as the psychological resources available to the individual, thereby including affective-emotional and cognitive dimensions. Associated with the promotion of psychological well-being are self-care practices, a pattern of activities initiated and carried out by individuals to preserve or improve their well-being and promote their health potential [22].

Satisfaction with academic life is a “multifactorial concept that encompasses all the students’ experiences related to their life in the academic environment, showing the quality of their adaptation to it” [23] (p. 71). Studies have presented academic life satisfaction as a predictor of psychological well-being [23,24,25]. Others have shown that satisfaction with life, while studying at higher education institutions, is linked to several factors, including happiness [26,27,28]. Moreover, academic success and resilience may be impacted by students’ perceptions of happiness and psychological well-being [27,28]. Therefore, satisfaction with academic life is a predictor of students’ psychological well-being, which may correlate with levels of MHL and MH outcomes, such as symptoms of anxiety and depression. Although there is no clear evidence of these relationships in university students, a recent study found that the MHL of postgraduate researchers is positively associated with well-being and help-seeking behavior and negatively associated with psychological distress [29].

Concerning the increase in stress, anxiety, and depression among students, it underscores the urgent need for targeted interventions that mitigate risk factors and strengthen protective factors, such as resilience and social support [11,30]. The prevalence of depression and anxiety among university health students is remarkably high, primarily likely due to the demanding academic curriculum, clinical rotations, and various personal stressors [31,32]. Self-care practices enable better strategies for managing daily stress, allowing for better management of personal life and daily stress, and this should be recognized as a strategy for improving MH [33].

This study has a convergent parallel mixed methods design and aims to assess MHL levels in university health students and explore its relationship with sociodemographic characteristics, academic situations, previous MH history, and psychosocial variables, such as academic satisfaction, happiness, psychological well-being, and psychiatric symptoms (quantitative data). Based on an integrated analysis of quantitative and qualitative data, this work also aims to identify knowledge gaps and educational needs regarding MHL, promoting personalized interventions that enable students to face emotional and academic challenges effectively.

With this in mind, the following research questions are proposed: (1) What are the levels of MHL in university students, and how are they related to sociodemographic characteristics, academic situations, previous MH history, and psychosocial variables? And (2) What are the main knowledge gaps and educational needs regarding MHL of these university students?

This study conceptualizes that MH problems in university students are on the rise and that low levels of MHL are related to adverse MH outcomes, and so it is predicted that: H1(a): Greater MHL levels will be associated with more positive MH outcomes.

## 2. Materials and Methods

### 2.1. Study Design

The current study is part of an ongoing wider research project called SMILE—“Student mental health literacy for improved academic life satisfaction, happiness, and psychological well-being”, financed by the Department for Research and Innovation of the higher education institution in the north of Portugal, where the study took place. This research project aims to implement a brief intervention tailored to specific students’ needs, who will be active agents in deciding on actions to promote their MH satisfaction with academic life and well-being.

This study used a convergent parallel mixed-methods design, as the simultaneous application of the quantitative (online cross-sectional survey) and qualitative [focus group (FG)] strands took place during the same phase of the research process, with the methods being given equal priority. After that, the quantitative and qualitative strands were analyzed independently (first quantitative data followed by qualitative data), followed by an integrated analysis to examine how the quantitative and qualitative results diverged, converged, or enhanced each other, ultimately providing a more holistic understanding of the MHL among university health students [34]. More specifically, in this study, an online cross-sectional survey (quantitative data) was carried out on the same day as the FG, allowing us to find out about university students’ levels of MHL and their relationship with sociodemographic and academic life characteristics and MH outcomes. FG interviews on the subject were then carried out with these participants. The survey data were analyzed quantitatively and the FG data qualitatively, and the two sets of findings were then integrated to assess divergence, convergence, or reinforcement.

The present study was based on the international guidelines of the Mixed Methods Article Reporting Standards (MMARS) [35].

**Figure 1:**
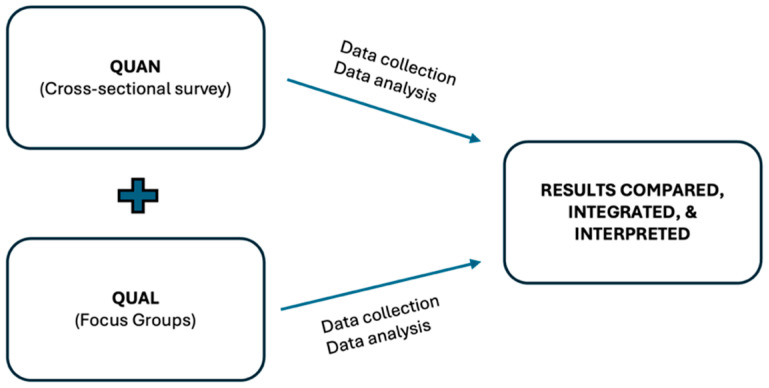
Study diagram of the convergent parallel mixed-methods approach.

The study diagram of the convergent parallel mixed-methods approach used is presented in Figure 1.

### 2.2. Ethics Considerations

The study was approved by the Research Ethics Committee of the higher education institution where the study took place (approval number: CE/IPSN/CESPU-48/24). Detailed information about the study was provided to the potential participants, and written informed consent was obtained from all participants. Participants also provided oral informed consent prior to the focus group (FG). The confidentiality of the data collected was protected through an anonymization process so as not to allow the identification of the participants.

### 2.3. Participant Recruitment and Data Collection

Email invitations were sent to university students (aged ≥ 18 years old, both sexes) who were attending one of the health degree studies at the Portuguese higher education institution in the north of the country, inviting them to participate in the study. If they accepted, a second email was sent with a link to access and fill in an online survey (quantitative data collection), as well as with the day and time of the three online FG (qualitative data collection). One follow-up email was sent to increase the participation rate (one week and two weeks after the initial email).

After agreeing to take part and obtaining informed consent, in the first phase, the participants were asked to fill in a cross-sectional online (quantitative data collection), using a research protocol that included a semi-structured questionnaire to record sociodemographics (e.g., age and sex), their academic situation (e.g., degree studies and year of study), and clinical data (e.g., family and personal history of mental illness), created specifically for this research project. This protocol also encompassed the following assessment instruments: MHL Questionnaire (MHLq-SVa) [36,37], Academic Life Satisfaction (ALS) [38], Subjective Happiness Scale (SHS) [39,40], Psychological Well-Being Scale (PWBS) [20,41], and Depression Anxiety Stress Scale (DASS) [42,43].

These participants were then integrated into the qualitative study. An FG guide interview was developed (please see Supplementary Material Table S1) with a total of eight open questions to explore the participants’ own understanding of the MHL concept, the MH challenges in higher education they face, and the characteristics of a targeted intervention addressing MHL promotion (qualitative data collection). The interview guide was developed based on questions on constructs such as those in the online survey and also the researchers’ literature review. The FG interviews were conducted by members of the research team with training in qualitative moderation (A.T. and T.M.) using the videoconferencing application Zoom. The FG sessions were recorded with the participants’ permission and then transcribed.

### 2.4. Data Analysis

The Statistical Package for the Social Sciences (SPSS) version 30.0 for Windows was used to perform the statistical analysis of the quantitative data collected in the online survey. A descriptive and association analysis between the MHLq-SVa (total and its four domains: Knowledge of mental health problems; Erroneous Mistaken beliefs/stereotypes; First aid and help-seeking skills; Self-help strategies) and the sociodemographic, academic (semi-structured questionnaire) and psychosocial variables of the study was carried out. For the descriptive analysis, frequencies and percentages for categorical variables and mean and standard deviation (SD) for continuous variables were used. The association between the quantitative variables in the study was analyzed using non-parametric tests since the data did not have a normal distribution. Thus, the Mann-Whitney U test and the Kruskal-Wallis tests were used for continuous variables at a significant level of 0.05. Spearman’s correlation coefficient was considered to evaluate the association between continuous variables. The internal consistency and reliability data of assessment instruments were assessed by using Cronbach’s alpha coefficient (α). A score ranging from 0.70 to 0.95 was considered acceptable for internal reliability [44,45].

For qualitative data, the content analysis proposed by Bardin [46] was performed, composed of the following phases: (a) organization of the analysis; (b) coding; (c) categorization; (d) treatment, inference, and interpretation of the results.

This process was conducted separately by two researchers (A.T. and S.M.), who subsequently met and discussed to achieve an agreement upon the organization of the content.

Selected quotes from the Portuguese transcript were translated, added in italics, and inserted in the following report. Codes from the participants were numbered according to the FG number (1…3) and by interviewee number (E…E24). The NVivo 15 software program was used for data management.

Regarding analyzing data integration, quantitative findings following qualitative results were described and analyzed separately, and then an integrated analysis was carried out by two researchers (A.T and S.M) and further reviewed by the other co-authors. Triangulation was considered in this study as a primary approach to integrating the results by systematically comparing, integrating, and interpreting the results of various data sources to better understand the research problem. More specifically, in this approach, the significant statistical quantitative results and the major themes in the qualitative findings were considered for merging to identify and discuss areas of convergence, divergence, or mutual reinforcement. This process resulted in the creation of a table representing the merging and interpretation of both data types [34,47].

## 3. Results

### 3.1. Quantitative Data Results

Data were collected over two months between June and July 2024. Twenty-four students participated in this cross-section survey study. The majority were female (75%), single (92%), and with a mean age of 25.5 (SD = 8.1). Around 67% were nursing students and 42% were in their third year. Regarding support needs for MH problems, 74% had had support from a service or health professional at some point in their lives, and about 17% were currently receiving support. Furthermore, 22% of participants were taking psychotropic medications, and 57% had a family history of mental disorders (Table 1).

The following statistically significant results were found when analyzing the association between the MHLq-SVa (total and domains) and other study variables.

In relation to sociodemographic data, women had a greater “knowledge of mental health problems” (MHL domain) compared to men (median = 28 vs. 25; *p* = 0.025).

Regarding MH history variables, higher scores in MHL total (median = 72 vs. 69.5; *p* = 0.020) and in the “self-help strategies” MHL domain (median = 19 vs. 18; *p* = 0.044) were identified among students with previous MH support needs. In addition, students with a family MH history presented a higher score in the “erroneous beliefs/stereotypes” domain of MHL (median = 15 vs. 13.5; *p* = 0.007). It is worth bearing in mind that a higher score in this domain (inverted items) indicated a lower level of MH erroneous beliefs/stereotypes. For more detailed information, please refer to Table S2 in Supplementary Material.

With respect to academic life satisfaction, a negative correlation between the MHL domain “knowledge of mental health problems” and satisfaction with the academic environment (r_s_ = −0.571; *p* < 0.01) was found in this study. No correlations were found between MHL and other assessed variables, including subjective happiness, psychological well-being, and psychiatric symptomatology (Table 2).

With regard to the internal consistency and reliability data of the assessment instruments, the Cronbach’s alpha coefficients for the obtained total and domains of each scale were overall considered acceptable (α > 0.70) [44,45], particularly in the instruments DASS, ALS, and SHS.

In more detail, the total and the domains of the MHLq-SVa revealed coefficients between α = 0.439 (First aid skills/seeking help domain) and α = 0.803 (Self-help strategies domain), which, although slightly lower, were close to those obtained in a previous validation study of this scale [37].

The Cronbach’s alpha coefficients for each dimension and the total ALS score were close, ranging from α = 0.79 to α = 0.81, and were similar to those found in the previous validation study [37]. In the SHS, a α = 0.80 for the total score was found, higher than those found in the previous validation study [40].

For PWBS, the Cronbach’s alpha coefficients for the total was 0.891, and for its six dimensions, the values ranged from α = 0.449 to α = 0.773. These values were slightly lower than those found in the validation of the Portuguese version of this scale [40], particularly in the domain of Positive Relations with Others (α = 0.449).

With regard to the DASS, the alpha values obtained for the total and domains (ranging from α = 0.944 to α = 0.832) were higher than those found in previous validation studies for this scale in Portugal [43].

### 3.2. Qualitative Data Results

A total of three FG interviews were carried out between June and July 2024, with a mean duration of 105 min (between 100 and 110 min). An average of eight students took part in each session. It was generally accepted that between four and twelve participants were sufficient for each FG [48].

From the FG content analysis, it was possible to explore the students’ perspectives of MHL, identify the needs they felt, and explore their characteristics, namely the topics to be addressed, the most favorable pedagogical strategies, and the resources to be mobilized to structure a brief intervention aimed at specific needs.

A total of five categories and twenty-seven subcategories emerged. In some of them, it was possible to identify secondary subcategories, enabling the authors to explore the students’ perception of the MHL-targeted intervention characteristics (please see Supplementary Material Table S3 for the categorization matrix).

#### 3.2.1. Concept

Regarding the MHL concept, students identified that MHL was more than knowledge about MH and led to a better understanding of it: *“Mental health literacy is a set of skills, a range, let’s say, of knowledge that leads us to an understanding and knowledge of mental health”* (F1E1). Also, it involved putting into practice and adopting behaviors favorable to the strategy’s adoption: *“…which will then also influence people’s attitudes and the way they recognise mental health and illness”* (F1E3). Students considered that the prevention related to the MHL concept had a major component: *“In this case, it’s really about focusing on prevention and action”* (F1E4).

#### 3.2.2. Intervention

For the development of a targeted intervention, students identified several characteristics. First, it needed to be linked with the course curriculum for the knowledge reinforcement:

*“…also integrate some things into the curricular units, because whether it seems like it or not, when we’re in the health field, it’s important to have this ability to have knowledge about what mental health is and I think it’s important throughout the year…”*.

(F2E9)

The group should be small (up to 15 students) with students from various health sciences degrees, ideally from other campuses, and be an open group where members rotate from session to session. From the students’ point of view, there are certain requirements for success, namely dissemination and their active engagement. The location should be close to each campus to avoid extra traveling, and it can take place in both face-to-face and online formats:

*“I think we could have a hybrid model, as we started by talking about being online, because it’s so easy. If from time to time a different dynamic comes up, with a different theme it could be face-to-face. We could do something like this, and then involve different groups, because in our case, as much as we socialise between different years, we’re all from the same course. So, we end up having a different perception and I can better understand the reality of my colleagues, who have a totally different reality to mine”*.

(F2E13)

There was no set number of sessions, only that they should take place throughout the semester, with a special focus on exams and internship periods. Each session should last between 45 min and 60 min:

*“I don’t think there necessarily has to be a number, because if there’s only one session it might not be enough. And so, I think, depending on the availability of the teachers or psychologists, I think it should be more or less planned throughout the semester, several sessions”*.

(F1E7)

The topics to be covered should go beyond knowledge about MH and disease prevention and focus on aspects related to training to mobilize coping strategies and resilience in order to deal effectively with day-to-day challenges and efficiently manage anxiety during periods of greater academic pressure: *“I think we could also emphasize and talk about coping strategies so that students can manage their moments of stress, their moments of anxiety, whether it’s a written test or a practical assessment”* (F1E5). All this, combined with managing their emotions, promoting self-knowledge, and strengthening skills to manage their relationships with peers: *“…it’s the emotional intelligence part, of being able to validate what you’re feeling”* (F2E9); *“…and I think it’s important to promote self-knowledge strategies”* (F3E17); *“Interpersonal relationships, with people, with friends…”* (F3E19).

It was important for them that the content address the beliefs and stereotypes surrounding MH in order to demystify preconceived ideas and be important for helping others and themselves: *“I think there’s a bit of a lack of demystification here. It’s also important to have these kinds of meetings where we discuss mental health, literacy and knowledge to break down some of the taboo ideas we have about mental health, mental illness and what these things are”* (F2E9); *“How can you approach the person? How can you help them? Strategies to help the person calm down in those moments? It’s something that’s not difficult, I think everyone should be able to do it, because at any moment someone gets into an anxiety crisis or a panic attack for whatever reason”* (F1E5).

To implement this intervention, the students suggested an active learning method, with case discussions, feedback, and the peer-sharing of ideas. On the other hand, they considered role-play and simulation to be an essential tool: *“I remember our simulations and that in reality there were cases that could be similar and that we would already have some interventions here or this line of reasoning that we could already act on. The fact that we had a role-play or a simulation of what we could do in that situation I think helped us later in our personal lives”* (F2E10).

#### 3.2.3. Caring for MH

All the participants recognized the importance of taking care of their MH and were able to list some strategies that they put into practice in their daily lives, namely self-care and leisure activities, but also strategies for emotional self-regulation and activities that promote well-being and balance between physical, mental, and psychological well-being in order to feel good about themselves:

*“…but health is also defined as complete physical, mental and psychological well-being and is a finding of a balance between the three dimensions. And if our mental health is affected, we won’t be in balance with our lives and everything around us. This goes hand in hand with what my colleague said about being in complete balance with all the dimensions of our lives”*.

(F2E14)

#### 3.2.4. Challenges in Higher Education

Students recognized that they face daily challenges to their MH in their academic context and environment, listing several: the environment itself, where socializing with peers is scarce and the context is just to attend classes; beliefs and stereotypes related to MH, but also the social role of the student and expectations of their performance; time and stress management, which are closely linked to the way in which a student will handle tasks and academic pressure in their day-to-day life: *“I know and often recognize that I took a long time to express what I was feeling or going through, out of a fear of being judged, and once I did express it, everything was fine. It’s just that we have this idea of being judged, and that I could be fine, but then I might come across as making a drama or something like that…”* (F2E10); *“We have a tendency, for example, to feel stressed, even though we have confidence in ourselves, in our knowledge, and feel prepared. There will always be that stress, and it will always have an influence”* (F1E1).

They also believed that there are activities, but their peers do not take part or seek them out, revealing a lack of interest and initiative: *“We give a warm welcome to first-year students, but they don’t show up, not even half of them, for example. In other words, you put in so much effort and care for their well-being, in terms of helping them start to adjust and create a sense of integration into higher education, but the students end up not wanting it”* (F2E12). They believed there is still a lack of MHL among peers and that it is necessary to improve not only knowledge but also promotion: *“… Exactly, it comes from the lack of mental health literacy, where people try to prevent illness rather than promote health. In other words, people focus on not becoming anxious or developing depression. This is often what is talked about in society, but they forget that for this to happen—or that this is different from having mental health—preventing a mental illness is not the same as actually having mental health. And I think there’s a lot of this lack of understanding”* (F3E22).

Although they identified strategies to take care of their MH, they recognized that there is still a lack of MH promotion: *“I think there is a lack of mental health promotion. In other words, we can try to take care of our mental health, but what’s missing is a sense of lightness, so to speak, in living our lives”* (F1E2).

Interpersonal relationships were valued by the students as a challenge, recognizing their importance, but acknowledging that at times there was a lack of understanding, closeness, and even empathy between them:

*“There were internship placements I had with a colleague where they were almost in paradise, everything was fine for them, while I felt like I just wanted it to end quickly and leave that place. In other words, even if I spoke to them, they would never be able to understand, and it ended up being a bit like what the colleague was saying—although not judging, they couldn’t comprehend my pain, so to speak. In the end, it felt like my experience was being devalued”*.

(F2E13)

#### 3.2.5. Institutional Resources

Regarding institutional resources, the students identified the existence of important resources that could be improved. Sports and leisure activities existed but could be better promoted: *“I think school sport would be important, I miss it a lot, in a way it helps us to prevent things from happening in the first place”* (F1E8).

They recognized the importance of the workshops and training that took place throughout the year as an added value to be maintained: *“…and it was very important that we had this relaxation and yoga workshop”* (F3E17).

The support and closeness to the teachers was very significant for them, reinforcing the importance of the pedagogical relationship as a social support: *“I think they give us a lot of confidence and make us feel at ease to deal with any problems we have. One way or another, I think it’s an asset”* (F2E10).

They recognized the counseling office as a resource they knew and knew how to use. However, they felt that its accessibility should be improved: *“More or less, because then there’s very little availability. I think there’s only one psychologist, so she has very little availability, usually from month to month”* (F2E14).

The institutional mentoring program was also mentioned by the students as an important asset for everyone: *“We have the mentoring program, the teachers themselves are part of the mentoring program. For example, I’ve been helping first-year students who came to me for their internship report, as a mentor”* (F1E7).

### 3.3. Data Integration and Triangulation Analysis

Table 3 displays the main quantitative and qualitative results separately, their integration with the analysis of convergence, divergence, or reinforcement.

## 4. Discussion

This study aimed to assess MHL levels in university health students and analyze their relationship with academic situations and psychosocial variables. Also, it aimed to identify knowledge gaps and educational needs regarding MHL, based on an integrated analysis of quantitative and qualitative data.

From the quantitative findings, MHL levels were particularly associated with some sociodemographic and MH history variables. More specifically, women had a greater knowledge of MH problems compared to men, as shown in the literature. Some studies revealed that women identified psychopathology symptoms more accurately than men [49,50]. In addition, students with previous MH support needs showed more MHL, as observed in the study of other authors [2], and MH self-help strategies. As described in the literature, students with MH history or who had required support for MH problems and experienced the symptoms had the awareness to recognize MH issues and know where to find appropriate support services [50,51]. Also, students with a family MH history presented lower levels of erroneous beliefs/stereotypes, which also reinforces the results found by Miles et al. [2]. Interestingly, students with greater knowledge of MH problems had less satisfaction with the academic environment, which was reinforced by the qualitative findings where these students identified necessary improvements in the academic environment in the adoption of strategies to promote MH. Recent studies have indicated that higher education institutions are uniquely positioned to implement educational initiatives, provide accessible psychological support services, and engage multidisciplinary teams to support students’ mental health needs [19].

Regarding the qualitative findings, it was possible to identify participants who highlighted the importance of self-care and leisure activities as essential to achieving a balance between physical, mental, and psychological well-being. Students emphasized that adopting daily practices to enhance well-being fosters a sense of control and improves their capacity to manage stress. The literature has proposed that efforts for balance between work and personal life, seeking sources of support, exercising, and getting adequate sleep are practices of self-care that promote well-being and MH [22]. In some, important are the self-regulation strategies and well-being practices to deal with and manage stress and adjust to the new academic environment. Programs that focus on self-regulation techniques, mindfulness, physical activity, and the development of coping mechanisms could be instrumental in supporting students as they navigate academic and personal challenges [32].

The academic environment for health students presents unique challenges not only at the time of entry but also throughout clinical training, which has a significant impact on students’ MH. Participants reported difficulties managing stress in an academic context, time management, and the high expectations associated with student performance, compounded by negative stereotypes about MH. These challenges often led to feelings of isolation and dissatisfaction with the academic environment. Zhang et al. [52], in a sample of medical college students, showed that psychological resilience decreased the level of psychological distress, and this relationship was partially mediated by MHL. These findings showed that efforts aimed at enhancing the MHL may prevent or reduce the prevalence of psychological distress symptoms among students.

These new challenges require the development of appropriate coping strategies and well-being practices for their peers, resilience, and social support in an academic environment that responds to the student’s needs. Feelings of engagement with the student community allow for a sense of belonging to the university and improve students’ overall well-being [28], as well as academic performance, less loneliness, and help-seeking [53].

Peer relationships also emerged as a factor identified by students, recognizing the importance of supportive interactions while highlighting the lack of understanding and empathy in some cases, especially MH. Strengthening peer networks and promoting inclusive dialogues around MH could reduce stigma [13,14] and create a more collaborative educational environment.

The qualitative results highlighted the importance of some institutional MH resources. Students expressed the need for more accessible, flexible, and engaging MH initiatives. They proposed hybrid models that combine online and face-to-face interventions, offering greater flexibility to accommodate students’ diverse needs. Active learning strategies, such as role-playing and simulations, have been identified as important in acquiring practical skills for dealing with self- and peer-reported MH issues, as mentioned by Reis et al. [50]. Students referred to the need to integrate MHL into the academic curriculum as a way of normalizing discussions around this topic and preparing students for future professional roles in health-related fields. Reis and colleagues [50] considered that integrating learning opportunities about MH into curriculum design could improve students’ MH and well-being.

Considering the demands of higher education on health, the students emphasized the importance of pedagogical, counseling, and mentoring programs. Recently, some authors have sought to enhance MHL in university, highlighting the positive interactions between student initiatives and counselor strategies [54]. These authors reported that counselor strategies aimed at enhancing students’ MHL involve counselors’ endeavors to boost students’ comprehension, awareness, and skills concerning their mental well-being and their surroundings [54]. Another important contribution was mentioned by Lo et al. [51], who suggested that interventions, such as Mental Health First Aid (MHFA), may potentially help clinical educators and health professional students to develop positive attitudes toward offering help and increasing MHFA knowledge. MHFA may reduce social distance from a person with a MH condition, but the content needs to be refined if they are to change attitudes toward seeking professional help or stigma [17].

Participants emphasized the importance of creating specific interventions during critical periods, such as exams and internships, when stress levels are usually high. These findings are in line with the literature, which suggests that timely, context-specific interventions can significantly reduce MH risks [11] and improve academic success.

In the present study, students identified that MHL involves putting knowledge about MH into practice and adopting behaviors that support the implementation of strategies. Students considered the prevention related to the MHL concept a major component because MHL helps them to recognize the signs and symptoms of mental problems, identify coping strategies, and seek help when needed, as reinforced by several authors [2,4,11,14,49,50,51,55].

## 5. Conclusions

The present study highlighted the importance of a systematic approach to merge the results by comparing, integrating, and interpreting the findings obtained from quantitative and qualitative data sources to better understand and explore MHL among university health students.

This study makes a relevant contribution to deepening knowledge about MHL among university students, as well as the factors associated with it. In addition, this work took into account various sociodemographic, psychosocial, and MH factors in the analysis of their relationship with MHL.

On the other hand, this study allowed us to explore not only the strategies used by students to promote their MH but also the domains that they consider to be relevant in the development of interventions focused on promoting their MH and MHL levels. In addition, the integration of MHL topics into the curriculum of the health professions was emphasized by the students.

All these results have future implications for the education of health professionals, practice, and research in this field. To be more specific, these findings contribute to the future development of a context-specific multicomponent level intervention that meets the needs of students to promote MHL in the academic environment by taking a holistic approach, which integrates risk factors and positive resources to enhance well-being and reduce distress in academic contexts. Moreover, the findings of this study reinforce the importance of adopting the PMeHL concept in new interventions that go beyond understanding MH problems, encompassing skills that promote well-being, personal fulfillment, and the ability to form social support networks. Additionally, this study seeks to contribute to the future development of replicable models that promote MH, reduce stigma, and create healthier, more collaborative educational environments.

However, this study has some limitations, namely due to the convenience sample recruited in a single context and gender imbalance, which requires some caution when interpreting the results. In addition, some restrictions on the size of the sample must be taken into account, particularly when analyzing quantitative data.

In line with the above, more research is needed to gain a better understanding of MHL in university students. It is recommended that multicenter, large-sample studies with a high level of evidence be carried out, which analyze, in depth, the MHL of university students from different sample groups and promote its development. Additionally, future studies should integrate other variables potentially related to MHL.

In this regard, the ongoing SMILE research project aims to continue investigating MHL in the context of university education, namely by identifying the predictors of low levels of MHL in university students, as well as defining and analyzing the preliminary efficacy of interventions that promote the MHL, and, consequently, the MH, the psychological well-being, and the satisfaction with academic life of these students.

## Figures and Tables

**Table 1:** Sociodemographic data, academic situation, and mental health history characterization of the sample (*n* = 24).

Age (years), mean (SD)	25.5 (8.07)
Sex, *n* (%)	
Male	6 (25.0%)
Female	18 (75.0%)
Marital Status, *n* (%)	
Single	22 (91.6%)
Married	1 (4.2%)
Divorced	1 (4.2%)
Degree Studies, *n* (%)	
Nursing	16 (66.7%)
Physiotherapy	8 (33.3%)
Year of Study, *n* (%)	
1st year	4 (16.7%)
2nd year	4 (16.7%)
3rd year	10 (41.6%)
4th year	6 (25.0%)
Previous MH support, *n* (%) (*n* = 23)	
Yes	17 (73.9%)
No	6 (26.1%)
Diagnosis of mental disorder/disease, *n* (%) (*n* = 21)	
Yes	4 (19.0%)
No	17 (81.0%)
Current MH support, *n* (%) (*n* = 23)	
Yes	4 (17.4%)
No	19 (82.6%)
Currently taking medication for anxiety, depression, or sleep problems, *n* (%) (*n* = 23)	
Yes	5 (21.7%)
No	18 (78.3%)
Nuclear family member with a mental illness or disorder, *n* (%) (*n* = 23)	
Yes	13 (56.5%)
No	10 (43.5%)

SD = standard deviation; MH = Mental Health.

**Table 2:** Correlations between MHL-q-SVa and other study variables.

	MHLq-SVa				
	Total	Knowledge of Mental Health Problems	First Aid and Help-Seeking Skills	Erroneous Beliefs/Stereotypes	Self-Help Strategies
ALS (*n* = 22)					
Total	−0.277	−0.373	0.075	−0.086	−0.198
Personal Satisfaction	0.034	0.029	0.118	0.110	−0.080
Satisfaction with the Academic Environment	−0.404	−0.571 **	0.135	−0.211	−0.188
SHS (total) (*n* = 22)	0.045	−0.105	0.410	0.113	−0.010
PWBS (*n* = 22)	0.045	−0.105	0.410	0.113	−0.010
Total	−0.122	−0.206	0.076	0.066	−0.012
Autonomy	−0.062	−0.111	−0.028	−0.380	0.310
Environmental Mastery	−0.215	−0.216	−0.076	0.071	−0.091
Personal Growth	0.214	0.188	0.120	0.076	0.179
Positive Relations with Others	−0.123	−0.106	0.283	0.202	−0.311
Purpose in Life	−0.047	−0.087	0.223	0.236	−0.120
Self-acceptance	−0.172	−0.335	0.100	0.234	−0.093
DASS (*n* = 21)					
Total	0.040	0.156	−0.147	−0.198	0.100
Depression	−0.118	0.000	−0.296	−0.308	0.018
Anxiety	0.069	0.190	−0.150	−0.072	0.078
Stress	0.082	0.181	−0.179	−0.144	0.147

MHLq-SVa—Mental Health Literacy Questionnaire; ALS—Academic Life Satisfaction; SHS—Subjective Happiness Scale; PWBS—Psychological Well-Being Scale; DASS—Depression Anxiety Stress Scale; ** *p* < 0.01.

**Table 3:** Data comparison, integration, and interpretation analysis.

Quantitative Results	Qualitative Results	Comparasion and Integration of Results	Convergence, Divergence, Reinforcement
Students with previous MH support needs showed more MHL (*p* = 0.020) and MH self-help strategies (*p* = 0.044).	Category: Caring for MH and Concept	Almost 74% of students reported previous support needs for MH problems, which was associated with more MHL and MH self-strategies. This is corroborated by qualitative data, with the emergence of the category Caring for MH, where students exposed many strategies for promoting and preventing MH. Also, students identified that MHL involves putting knowledge about MH into practice and adopting behaviors favorable to the strategy’s adoption.	Convergence
Students with family MH history presented lower levels of erroneous beliefs/stereotypes (*p* = 0.007).	Category: Challenges in Higher Education—Subcategory: Beliefs and stereotypes	Half of the sample had a nuclear family member with a history of mental illness. These students also showed fewer stereotypes about mental illness. These data were reinforced by the subcategory Beliefs and stereotypes, which showed that students had a perception of what contributes to stigma and discrimination around MH and illness.	Reinforcement
Students with higher knowledge of MH problems had less satisfaction with the academic environment (r_s_ = −0.571; *p* < 0.01).	Category: Challenges in Higher Education—Subcategories: Academic environment; Interest and initiative Category: Institutional resources	The association between greater knowledge of MH problems and lower satisfaction with the academic environment can be partly explained by these students perceiving that the academic environment does not provide the appropriate conditions for promoting MH. This is reinforced by the category about existing resources in the institution, which comprised suggestions from students for strategies to promote MH at an institutional level.	Reinforcement

MHL = Mental Health Literacy; MH = Mental Health; *p* = *p*-value; r_s_ = Spearman’s rank correlation coefficient.

## Data Availability

The data presented in this study are available on request from the corresponding author.

## Supplementary Materials

The following supporting information can be downloaded at: https://www.mdpi.com/article/10.3390/healthcare13070724/s1, Table S1 provides more detailed information of the non-parametric statistical analysis carried out. The focus group guides are presented in Table S2. Table S3 provides the categorisation matrix.

## Abbreviations

The following abbreviations are used in this manuscript:

MH	Mental health
MHL	Mental health literacy
PMeHL	Positive MHL
MMARS	Mixed Methods Article Reporting Standards
FG	focus group
MHLq-SVa	MHL Questionnaire
ALS	Academic Life Satisfaction
SHS	Subjective Happiness Scale
PWBS	Psychological Well-Being Scale
DASS	Depression Anxiety Stress Scale
MHFA	Mental Health Fist Aid
