# Supplementary material for: A Mixed Methods Approach to Understanding Mental Health Literacy Among University Health Students
Source: Healthcare (Basel). 2025 Mar 25;13(7):724. doi: 10.3390/healthcare13070724 (PMC11989114; doi:10.3390/healthcare13070724)
Supplement: Supplementary file 1 [file healthcare-13-00724-s001.zip › Supplementary Materials_Table S1.pdf]

Supplementary Materials

Table S1. Focus Group guide interview

	Questions
1)	What do you understand by MHL?
2)	What is the importance of mental health care? How do you look after your mental health?
3)	What resources exist in the institution to promote or address mental health?
4)	What are the relevant topics/themes for a MHL promotion intervention for higher education students?
5)	What is the most appropriate number of sessions and length for developing this psychoeducational intervention?
6)	What are the most appropriate methods, pedagogical techniques and teaching resources for developing this psychoeducational intervention?
7)	What requirements are there for this psychoeducational intervention to be feasible?
8)	Is there anything else you think is important to share?
