# Supplementary material for: A Mixed Methods Approach to Understanding Mental Health Literacy Among University Health Students
Source: Healthcare (Basel). 2025 Mar 25;13(7):724. doi: 10.3390/healthcare13070724 (PMC11989114; doi:10.3390/healthcare13070724)
Supplement: Supplementary file 1 [file healthcare-13-00724-s001.zip › Supplementary Materials_Table S2.pdf]

## Supplementary Materials

**Table S2**. Detailed results of the non-parametric statistical analysis (n=24)

| MHLq-SVa | Total | | Knowledge of mental health problems | | First aid and help-seeking skills | | Erroneous beliefs/stereotypes | | Self-help strategies | |
|---|---|---|---|---|---|---|---|---|---|---|
| | Mdn (m-M) | p-value | Mdn (m-M) | p-value | Mdn (m-M) | p-value | Mdn (m-M) | p-value | Mdn (m-M) | p-value |
| Sex, n (%) | | | | | | | | | | |
| Male | 71(65-75) | 0.359* | 25(22-26) | **0.025*** | 13(12-15) | 0.514* | 15(13-15) | 0.604* | 18.5(16-20) | 0.943* |
| Female | 71(67-79) | | 28(23-30) | | 14(11-15) | | 15(12-15) | | 18(13-20) | |
| Marital Status, n (%) | | | | | | | | | | |
| Single | 71(65-79) | 0.509* | 26(22-30) | 0.470* | 14(11-15) | 0.734* | 15(12-15) | 0.152* | 18(13-20) | 0.239* |
| Other (married or divorced) | 74(71-77) | | 27.5(25-30) | | 13.5(13-14) | | 13.5(13-14) | | 19.5(19-20) | |
| Field of Degree, n (%) | | | | | | | | | | |
| Nursing | 71(65-79) | 0.474* | 26(22-30) | 0.374* | 14(11-15) | 0.462* | 15(12-15) | 0.397* | 18(13-20) | 0.894* |
| Physiotherapy | 70(67-78) | | 25(23-30) | | 14(12-15) | | 14.5(12-15) | | 18.5(16-20) | |
| Course year, n (%) | | | | | | | | | | |
| 1st year | 70.5(65-77) | 0.617** | 24.5(22-28) | 0.168** | 12.5(12-14) | 0.253** | 15(13-15) | 0.327** | 18.5(18-20) | 0.262** |
| 2nd year | 72(68-77) | | 26(25-29) | | 12(12-15) | | 15(15-15) | | 18(16-19) | |
| 3rd year | 73(69-79) | | 28(23-30) | | 14(11-15) | | 14(12-15) | | 19.5(13-20) | |
| 4th year | 70.5(67-78) | | 25(23-28) | | 14(13-15) | | 15(13-15) | | 17.5(16-20) | |
| Previous mental health support, n (%)(n=23) | | | | | | | | | | |
| Yes | 72(67-79) | **0.020*** | 28(23-30) | 0.059* | 14(11-15) | 0.468* | 15(12-15) | 0.338* | 19(13-20) | **0.044*** |
| No | 69.5(65-71) | | 25(22-26) | | 13.5(12-15) | | 14(12-15) | | 18(16-18) | |
| Mental disorder diagnosis, n (%)(n=21) | | | | | | | | | | |
| Yes | 74.5(70-79) | 0.148* | 27(24-30) | 0.466* | 13(12-14) | 0.309* | 15(15-15) | 0.099* | 19.5(19-20) | 0.067* |
| No | 71(65-78) | | 25(22-30) | | 14(11-15) | | 15(12-15) | | 18(13-20) | |
| Current mental health support, n (%)(n=23) | | | | | | | | | | |
| Yes | 71(67-78) | 0.838* | 25.5(25-28) | 0.836* | 13(12-15) | 0.449* | 15(13-15) | 0.579* | 18(16-20) | 0.677* |
| No | 71(65-79) | | 26(22-30) | | 14(11-15) | | 15(12-15) | | 18(13-20) | |
| Currently taking psychotropic medication, n (%)(n=23) | | | | | | | | | | |
| Yes | 77(70-79) | 0.060* | 28(25-30) | 0.074* | 14(14-15) | 0.263* | 15(14-15) | 0.234* | 20(13-20) | 0.421* |
| No | 71(65-78) | | 25(22-30) | | 13.5(11-15) | | 15(12-15) | | 18(16-20) | |
| Nuclear family member with mental disorder, n (%)(n=23) | | | | | | | | | | |
| Yes | 72(67-79) | 0.399* | 26(24-30) | 0.411* | 14(12-15) | 0.630* | 15(13-15) | **0.007*** | 19(13-20) | 0.799* |
| No | 71(65-78) | | 25.5(22-30) | | 14(11-15) | | 13.5(12-15) | | 18(16-20) | |

Notes: Mdn=Median; m= Minimum; M=Maximum; MHLq-SVa= Mental Health Literacy Questionnaire; *Mann-Whitney Test; ** Kruskal Wallis Test; The bold characters in the p-Value column refer to the significant p-values ($p<0.05$)
