# Supplementary material for: A Mixed Methods Approach to Understanding Mental Health Literacy Among University Health Students
Source: Healthcare (Basel). 2025 Mar 25;13(7):724. doi: 10.3390/healthcare13070724 (PMC11989114; doi:10.3390/healthcare13070724)
Supplement: Supplementary file 1 [file healthcare-13-00724-s001.zip › Supplementary Materials_Table S3.pdf]

Supplementary Materials

Table S3. Categorization matrix

Theme	Category	Subcategory	Subsubcategory
MHL	Concept	Behaviour and attitude	
		Knowledge	
		Comprehension	
		Prevention	
	Intervention	Link with curricula	
		Group features	
		Requirements	Dissemination
			Student engagement
			Location
			Format
		Timetable and time frame	
		Topics	Coping strategies and resilience
			Beliefs and stereotypes
			MH promotion
			Emotional intelligence
			First aid
		Pedagogical strategy	Active learning
			Workshop
			Role-play and simulation
Mental Health	Caring for MH	Leisure activities	
		Self-care	
		Self-regulation	
		Well-being	
	Challenges in higher education	Academic environment	
		Beliefs and stereotypes	
		Time management	
		Stress management	
		Interest and initiative	
		MHL	
		MH promotion	
		Peer relationships	
	Institutional resources	Sports and leisure activities	
		Education and training	
		Pedagogical relationship	
		Counselling service	
		Mentoring programme	
